# Long-term three-dimensional skeletal effects of hybrid hyrax with facemask versus mentoplate in growing Class III patients: a randomized controlled trial

**DOI:** 10.1186/s40510-025-00561-7

**Published:** 2025-04-21

**Authors:** Joeri Meyns, Jindanil Thanatchaporn, Sohaib Shujaat, Constantinus Politis, Reinhilde Jacobs

**Affiliations:** 1 Ziekenhuis Oost-Limburg, Genk, Belgium; 2 KU Leuven, Leuven, Belgium; 3 Chulalongkorn University, Bangkok, Thailand; 4 King Abdullah International Medical Research Center, College of Dentistry, King Saud Bin Abdulaziz University for Health Sciences, Ministry of National Guard Health Affairs, Riyadh, Kingdom of Saudi Arabia; 5 Karolinska Institutet, Stockholm, Sweden

## Abstract

**Background:**

Early intervention in Class III malocclusion aims to prevent the need for surgery in adulthood by enhancing upper jaw growth while limiting lower jaw development. Although traditional facemask treatment remain common, bone-anchored devices are increasingly used, claiming better skeletal control and patient compliance. However, strong evidence supporting these advantages is limited.

**Methods:**

Single-center, parallel-group, randomized controlled trial with 1:1 allocation ratio. *Participants:* 28 growing Class III patients (mean age 9.7 ± 1.3 years) in mixed dentition with skeletal class III malocclusion. *Interventions:* Patients were randomly assigned to either hybrid hyrax with facemask (HH + FM, n = 14) or hybrid hyrax with mentoplate (HH + MP, n = 14). All received Alt-RAMEC protocol expansion. FM group used 360–400 g/side elastic traction 12–14 h daily; MP group used 185 g/side continuous traction. *Objective:* To compare 5-year three-dimensional (3D) skeletal effects between HH + FM and HH + MP protocols. *Outcome:* Primary outcome was 3D volumetric changes of upper and lower jaw at 1 year (T1) and 5 years (T2) post-treatment, measured using low-dose CT scans. Randomization: 28 patients were allocated to either treatment-protocols using sequentially numbered opaque, sealed envelopes. The randomization sequence was generated with a 1:1 allocation ratio. *Blinding:* Due to the nature of the trial, the operator and children could not be blinded to the treatment allocation. However, blinding was used when assessing the outcomes.

**Results:**

Follow-up: one patient was lost at the one-year follow-up and an additional three patients were lost at the 5-year-follow-up. Outcomes: At T2 (5 years), maxillary advancement was identical between both groups (0.85 mm ± 0.5). Mandibular growth control showed minimal difference (FM: − 0.01 mm ± 0.24; MP: 0.10 mm ± 0.33). No significant differences were found between groups for any skeletal measurements (*p* > 0.05). Male patients showed lager mandibular changes both signed (*p* < 0.03) and unsigned (*p* < 0.01). Harms: minor harms were encountered with the anchor hooks (fracture or mucosal irritation), however none led to treatment cessation.

**Conclusions:**

Both protocols demonstrated comparable long-term skeletal effects in Class III correction. Treatment choice should be based on individual patient factors rather than assumed mechanical advantages.

**Supplementary Information:**

The online version contains supplementary material available at 10.1186/s40510-025-00561-7.

## Introduction

### Background

Class III malocclusion affects approximately 0–26% of the population [1] and can significantly impact both function and aesthetics. While early intervention is often recommended, clinicians face a critical choice: should they use traditional facemask (FM) therapy [2] or newer bone-anchored techniques [3]? Despite the growing popularity of bone-anchored treatments, no long-term randomized trials have compared their effectiveness to traditional approaches [4–6]. While FM therapy is the traditional treatment approach with well-documented results, its long-term success is not guaranteed. Most studies on interceptive class III treatment typically have limited long-term follow-up data [4]. Treatment effects often diminish over time as patients continue to grow. Limited long-term studies indicate that 25–30% of patients may still require orthognathic surgery despite early intervention [7, 8]. Additionally, traditional FM therapy can cause unwanted effects, including mandibular autorotation and dental compensations, primarily because forces are applied indirectly through the teeth [9]. To overcome the limitations of conventional FM therapy and address more severe class III deformities, various skeletal anchorage techniques, using screws and bone plates, have gained popularity and are used in conjunction with both intra- and extra-oral devices [10–24]. Despite the widespread use of skeletal anchorage devices for interceptive Class III treatment, there is ongoing debate about their effectiveness compared to traditional FM treatment [4–6, 12, 25, 26]. High-quality comparative evidence, particularly randomized controlled trials (RCTs), is scarce in this field [4–6, 26]. While skeletal anchorage techniques offer advantages, they are not without potential drawbacks, such as the need for invasive procedures and potential instability of anchoring devices [15, 27].

The hybrid hyrax (HH) device [28] utilizes two mini-implants in the anterior palate to provide skeletal anchorage for maxillary protraction while also performing rapid maxillary expansion. Although the effectiveness of rapid palatal expansion (RPE) in enhancing maxillary protraction, remains controversial [29–31], HH therapy is gaining popularity due to its low invasiveness compared to traditional bone-anchors in the upper jaw. However, its effectiveness when combined with various protraction methods, such as FM and mentoplate (MP), has not been directly compared in a controlled study. Moreover, no comparative three-dimensional (3D) data exists quantifying the skeletal changes following HH + FM and HH + MP treatment protocols [4]. The potential advantages of MP over FM in terms of skeletal impact and patient compliance have been hypothesized, but not empirically verified in a comparative study [25].

### Objective

This prospective RCT aimed to compare the 5-year long-term 3D skeletal effects of HH + FM and HH + MP treatment protocols in growing class III patients using low dose computed tomography (CT) scans. A previously published study by the same authors was published in the European Journal of Orthodontics, which reported on the 2D cephalometric changes of the same patient cohort [32].

## Methods

### Trial design

A single-center 2-arm parallel randomized controlled trial with 1:1 allocation ratio.

### Participants

This study examined patients with Class III skeletal malocclusion that were referred to our hospital by their orthodontist. Enrollment took place from December 2016 to September 2018 (complete data available in Supplementary File 1). All participants were in mixed dentition with anterior crossbite or an end-to-end incisor relationship and Class III molar relationship at the start of treatment. Patients were excluded if they had cleft/craniofacial syndromes, prior orthodontic/surgical treatment, significant skeletal asymmetry, or functional Class III malocclusion. No agenesis of a permanent central incisor was present in any patient. A single surgeon performed HH screw and MP placements, while three experienced orthodontists provided orthodontic care.

### Interventions

- Palatal expansion by HH

**Fig. 1:**
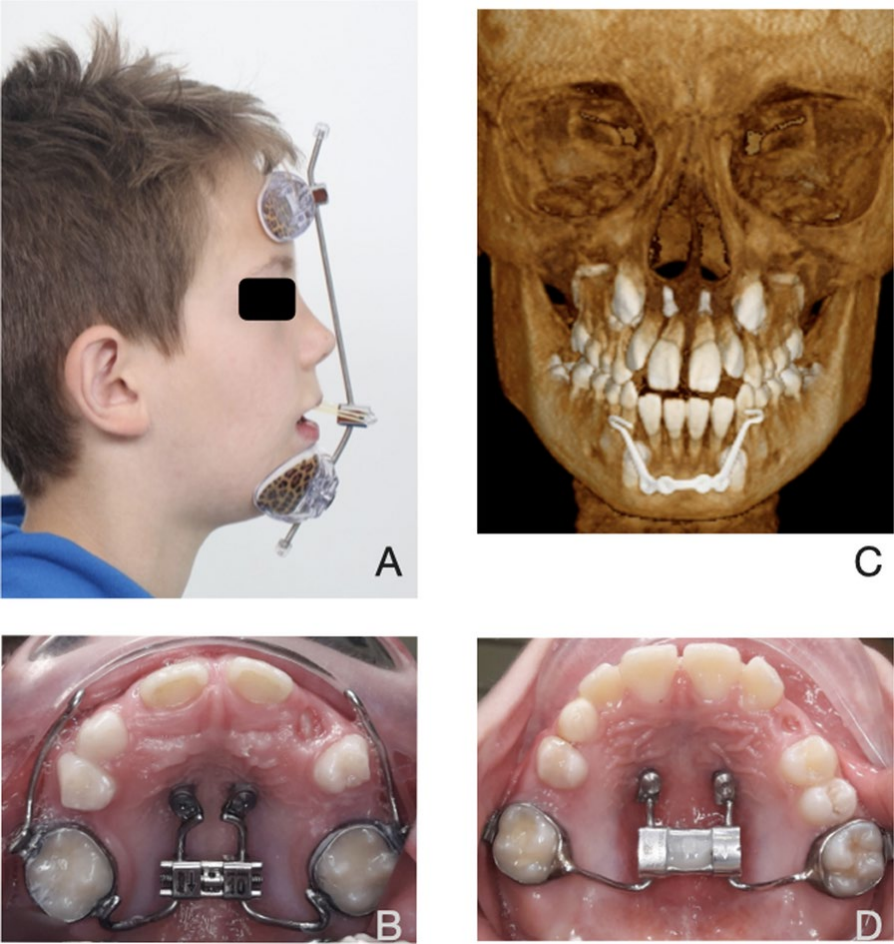
**A** extra-oral view Facemask (FM), **B** Hybrid hyrax FM, **C** Mentoplate (MP) secured between impacted canines, **D** Hybrid hyrax MP. Reproduced from Meyns et al. [32] with authors’ permission

In all patients, 2 self-drilling mini-screws (2 mm in diameter and 9 mm long, Benefit miniscrews, PSM-medical solutions®, Gunningen, Germany) were inserted free-handed into the anterior palate around the third rugae. A HH device was constructed with an expansion screw (Forestadent®, Pforzheim Germany) and welded to bands. These bands were then cemented to the first upper molars with a light cured cement (Band-Lok®, Reliance Orthodontic Products, Thorndale, USA) (Fig. 1). The Alternate Rapid Maxillary Expansion and Constriction (Alt-RAMEC) protocol [33] was implemented, where the hyrax was activated by the patient’s parents twice a day (0.25 mm per turn, two turns in morning and two turns at night) for 1 week, then it was deactivated twice a day (two turns in morning and two turns at night) for 1 week. This alternating activation and deactivation cycle was carried out three times. Subsequently, in the ensuing week, the maxilla was adjusted to the suitable transverse dimension.

b. FM group

In this group, FM therapy commenced concurrently with Alt-RAMEC. Elastics (American Orthodontics®, Sjeboygan, USA) were connected from the hooks of the expander to the FM (Orthocomfort & Medical Distributors SL®, Barcelona, Spain), creating a downward and forward vector. This configuration produced orthopedic forces of 360–400 g per side (equivalent to 12.7–14.0 oz). The patients were instructed to use the device for 12–14 h daily, primarily overnight, for the initial 6 months or until they achieved a positive overjet of at least 2 mm. For the following 6 months, the FM was to be worn only during sleep (Fig. 1).

c. MP group

During general anesthesia, MP (PSM-medical solutions®, Gunningen, Germany) was inserted through a marginal gingival incision. The plate was bent and modified before fixation with 2–4 screws (KLS Martin®, Tuttlingen, Germany) (Fig. 1). The patients were simultaneously given Alt-RAMEC and protraction elastics, generating orthopedic forces of 185 g per side (equivalent to 6 ½ oz). Elastic traction was initiated approximately 2 weeks after plate insertion, once the soft tissues had healed. Full force was applied immediately, without gradual buildup. The patients were instructed to wear it continuously, 24 h a day, 7 days a week, including during meals. They were also advised to replace the elastics daily for the first 6 months or until a positive overjet of at least 2 mm was achieved. For the subsequent 6 months, the elastics were to be worn only during sleep.

d. Fixed appliances

Phase 1 consisted of protraction therapy alone, without fixed appliances, from baseline (T0) to 1 year (T1). In Phase 2, all patients received standard fixed orthodontic appliances (edgewise mechanics, MBT 0.022, Empower R, American Orthodontics ®). Both phases followed standardized treatment protocols. In three MP patients and one FM patient, four premolar extractions were required. Additionally, one FM patient underwent the extraction of two premolars in the lower jaw.

### Outcomes

The primary outcomes were 3D volumetric changes of upper and lower jaw at T1 and T2 post treatment time-points.

- Radiographic data acquisition

Low-dose CT scans were acquired by the same technician at three time points (T0, T1, and T2). All patients were positioned supine in the CT scanner, with a wax bite in centric relation placed between their teeth in first tooth contact. Subjects were given instructions to remain still, abstain from swallowing, and maintain normal breathing during the scan acquisition. Scans were performed using a Somatom Force dual-source dual-energy CT system (Siemens®, Erlangen, Germany) with the following parameters:—0.6 mm slice thickness—0.3 mm increment—1.0 pitch—200 × 200 mm field of view—150 kVp tube voltage. CT scans delivered radiation doses of 0.095–0.257 mSv, similar to cone-beam CT (CBCT) levels. The CT system provided superior image quality and faster scanning times, reducing motion artifacts while maintaining low radiation exposure through automatic dose modulation.

b. 3D skeletal analysis

**Fig. 2:**
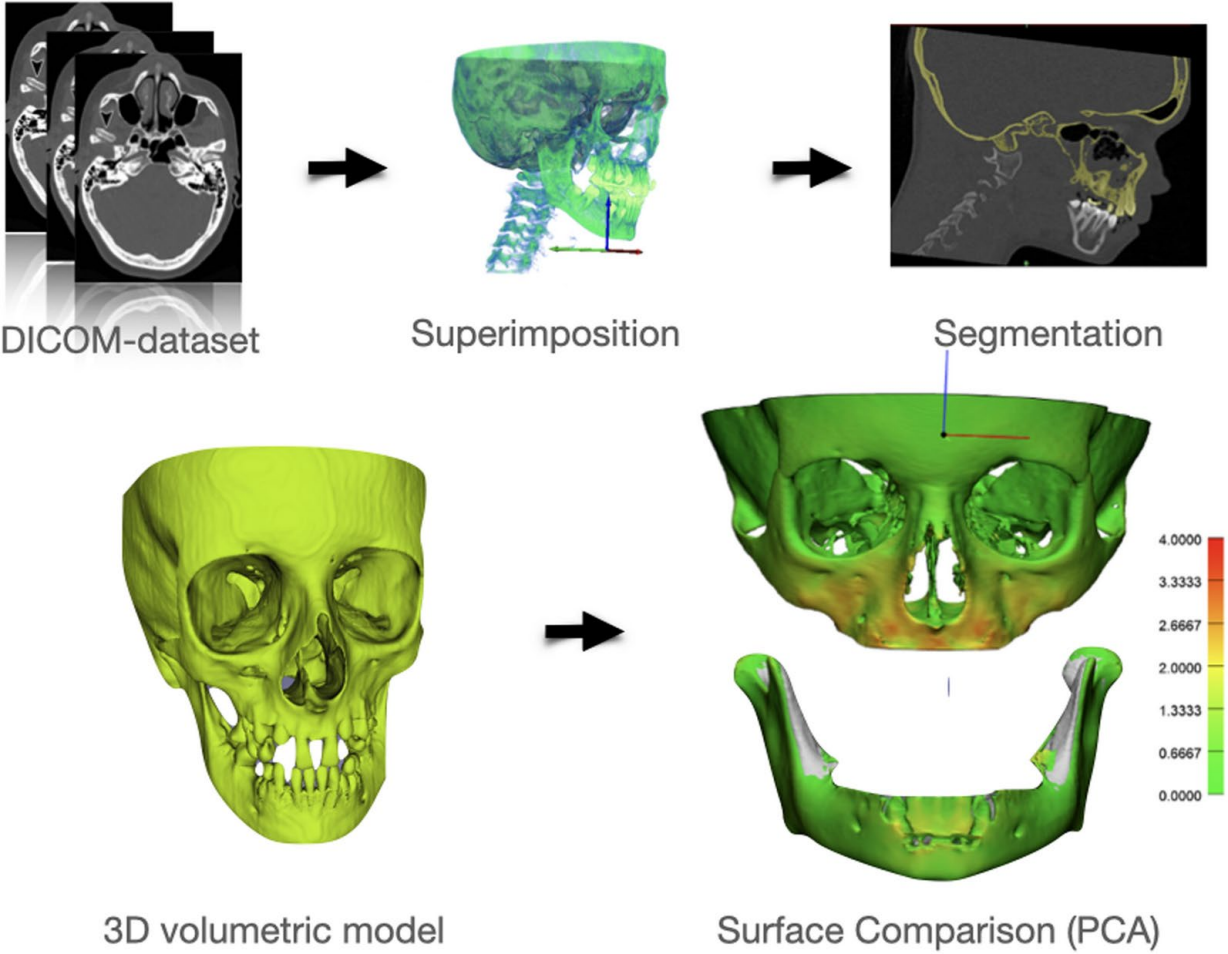
Flow-diagram 3D volumetric analysis: (i) Superimposition of T0/T1/T2 data-set in Amira® (maxilla superimposed on cranial base, mandible superimposed on internal symphyseal region); (ii) Segmentation in Mimics® generating 3D volumetric models (*stl); (iii) Surface comparison through Part Comparison Analysis (PCA) in 3-matic® (Teeth and surrounding alveolar bone are excluded)

The T0, T1 and T2 CT datasets were imported into Amira® software (version 2019.1, Thermo Fischer Scientific®, Merignac, France) in DICOM format. Volume rendering was applied to visualize the skeletal tissue. A rigid voxel-based registration using mutual information was performed to superimpose the T1 and T2 onto T0 datasets. The maxilla and mandible were separately registered using the anterior cranial base for the maxillary region and the anterior chin and internal symphysis for the mandible, as these regions are recognized as stable and reliable for voxel-based superimposition in growing patients [34–36]. The registered CT datasets were then imported into Virtual Patient Creator (version 2.2.0, September 2024, Relu ® BV, Leuven, Belgium), an online cloud-based platform that enables automatic segmentation of the maxillofacial anatomical structures. The anatomical segmentation, including both the maxillary and mandibular bones, was exported in Standard Tessellation Language (STL) format and refinement was performed in Mimics® software (version 20.0, Materialise®, Leuven, Belgium), if necessary. Teeth and surrounding alveolar bone were excluded from measurements to isolate true skeletal changes. The refined 3D surface models were imported in to 3-matic® software (version 14.0, Materialise®, Leuven, Belgium) for surface distance measurement between T0 and registered T1/ T2 maxilla and mandible. This allowed for the automatic calculation of the treatment effect, as mean difference between T0/T1, T0/T2 and T1/T2 images which was represented by a color-coded map. Figure 2 illustrates the flowchart outlining the methods, starting from CT registration to the final analysis. All measurements were performed by two independent operators (SS and TJ), and 10% of the data were analyzed twice to determine the inter- and intra-observer reliability.

c. Cephalometric analysis of baseline values

The assessment of Class III severity was conducted by evaluating the ANB angle and Wits’ appraisal at the commencement of treatment. This evaluation was performed on two-dimensional (2D) cephalometric image, generated from the CT dataset, with Planmeca Romexis® software (version 6.3.0, Planmeca®, Helsinki, Finland), using an orthogonal method without magnification. Cephalometric analysis was done with the OnyxCeph® software (version 3.6, Image Instruments GmbH®, Chemnitz).

The skeletal maturation was assessed according to the cervical vertebral maturation method [37]. Two independent oral and maxillofacial surgery trainees analyzed 10% of the data twice to determine the inter- and intra-observer reliability.

### Sample size calculation

When this trial began in 2016, no comparative studies of these specific techniques existed. In 2010 Cevidanes et al. [11] conducted a controlled clinical trial comparing Bone Anchored Maxillary Protraction (BAMP) with Facemask and Rapid Maxillary Expansion (FM-RME), finding a mean Wits difference of 2.3 mm between groups. We pooled the standard deviations from both groups by first converting them to variances by squaring the standard deviations, then taking the average, and converting the average back to a standard deviation by taking the square root (resulting in a SD = 2,0). A sample size calculation was performed for a one-sided t-test with a significance level of 0.05 and a power of 80%. This resulted in a required sample size of 12 patients per group. R version 4.1.2 was used, with the TrialSize library to calculate the sample size. We slightly overrecruited to account for potential dropouts.

### Randomisation

- Sequence generation

The randomization sequence was generated with a 1:1 allocation ratio. (for complete data, supplementary material 1).

b. Allocation concealment

Sequentially numbered sealed, opaque envelopes.

c. Implementation

The envelopes containing the allocation sequence codes were given to the patient by an intermediary and opened sequentially at the time of enrollment, excluding the clinician from the process.

### Blinding

Due to the nature of the trial, the operator and children could not be blinded to the treatment allocation. However, blinding was used when assessing the outcomes. This was achieved by pseudonymizing all patient data before and after treatment. The statistician analyzing the results was unaware of the group assignments.

### Statistical methods

We used a generalized estimating equation (GEE) model to assess treatment effects. The model analyzed differences between time points (T5–T0, T1–T0, and T5–T1), with measurements clustered by patient. This approach accounts for measurement error while incorporating all data points. The model included treatment group interactions across time points, along with covariates for patient age (in months), gender, and gonial angle. An identity link function was applied to handle normal responses, similar to linear regression but adjusted for clustered data. We specified an unstructured working correlation matrix and implemented the model using the geeglm function in R (version 4.4.1) from the geepack package. Reliability of cephalometric and 3D volumetric measurements was assessed using intra-class correlation coefficients (ICC) with 95% confidence intervals. Statistical significance was set at *p* < 0.05. Cephalometric measurements at baseline are described in mean with standard deviations. Unpaired two-sided samples t-test was employed for comparisons between the two groups.

## Results

### Participants flow

**Fig. 3:**
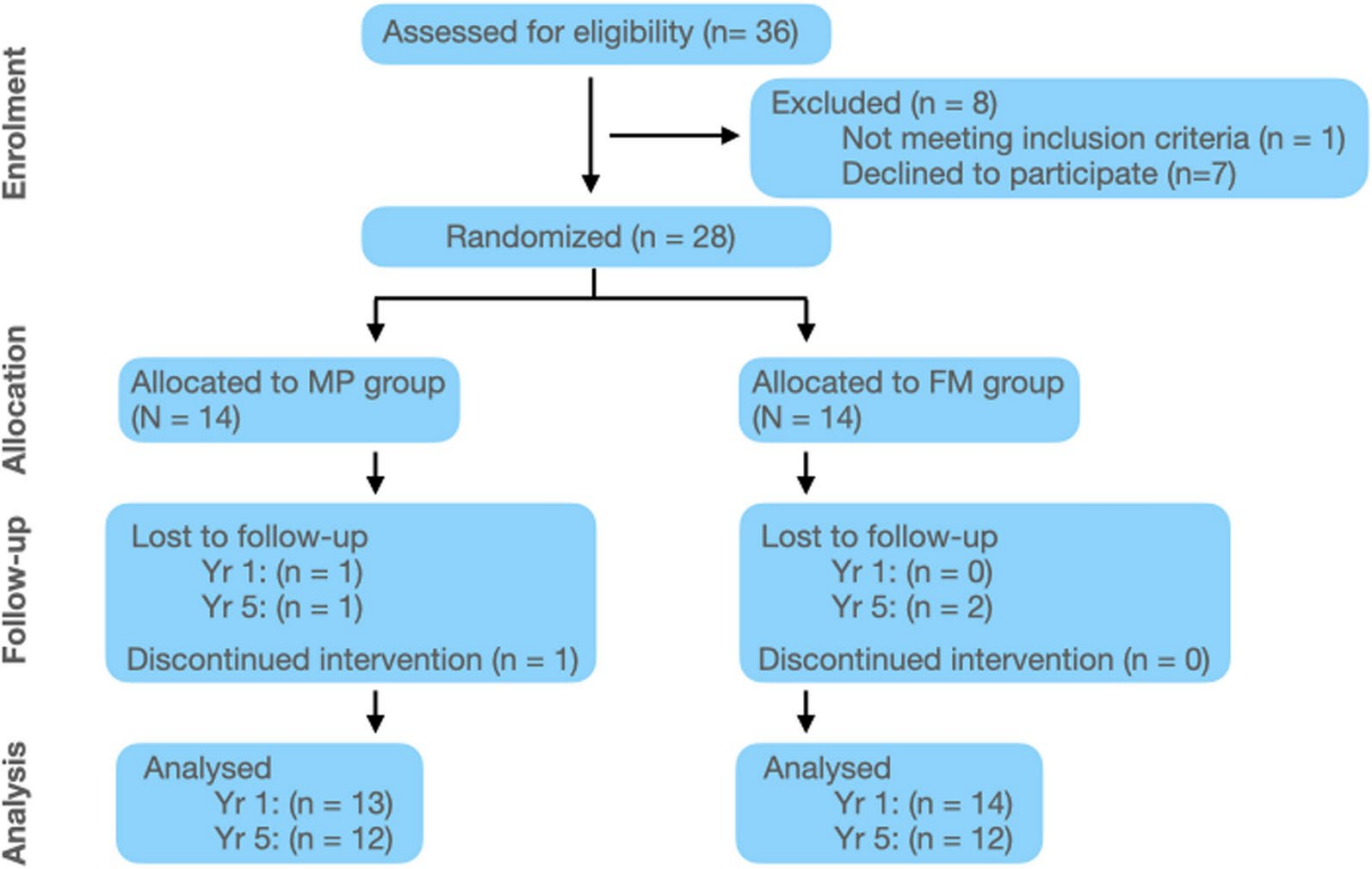
Participants flow. Reproduced from Meyns et al. [32] with authors’ permission

A total of 28 patients were recruited, comprising of an equal number of males and females (n = 14 each). One patient, initially assigned to the MP group, was later excluded due to non-cooperation leading to discontinuation of treatment. Three additional patients, two from the FM group and one from the MP group, were lost to follow-up before the 5-year time-point (Fig. 3).

### Recruitment

Patient recruitment occurred from December 2016 to September 2018. Scans were performed in three phases: initial scans (T0) from February 2017 to September 2018, first follow-up (T1) from February 2018 to August 2019, and second follow-up (T2) from October 2022 to February 2024. (Supplementary material 1 for more details).

### Baseline data

The study included 28 patients (14 of each gender, average age 9.7 years ± 1.3). Both treatment groups were matched for age and gender. All but one patient had reached cervical vertebral maturation stages 2 or 3. Both groups showed similar Class III malocclusion severity based on ANB and Wits measurements (Table 1).

**Table 1:** Group characteristics at T0 (start treatment)

	Facemask	Mentoplate	*p*-value
	Mean (SD)	Mean (SD)	
Sagittal			
SNA	79.41 (2.43)	78.38 (3.50)	0.38
SNB	80.22 (2.14)	78.94 (4.25)	0.33
ANB	− 0.82 (2.03)	− 0.55 (1.45)	0.69
Wits (mm)	− 5.71 (1.82)	− 5.62 (2.36)	0.90
F/M	7/7	7/7	
Age (T0)	9 y, 6 mo	9 y, 7 mo	
Follow-up (T2)	64 mo	65 mo	

F/M, Female/Male; Follow-up (T2), mean follow-up time in months, *p*-value (unpaired two-sided samples t-test). Baseline comparisons are provided for descriptive purposes only, to confirm group equivalence following randomization, and should not be interpreted as inferential statistical analyses. Reproduced from Meyns et al. [32] with authors’ permission

### Numbers analyzed

Twenty-eight patients (14 FM, 14 MP) underwent low dose CT at baseline (T0). At T1, 27 patients (14 FM, 13 MP) completed follow-up scans. By T2, 24 patients (12 FM, 12 MP) remained for analysis (Fig. 3).

### Outcomes and estimation

**Fig. 4:**
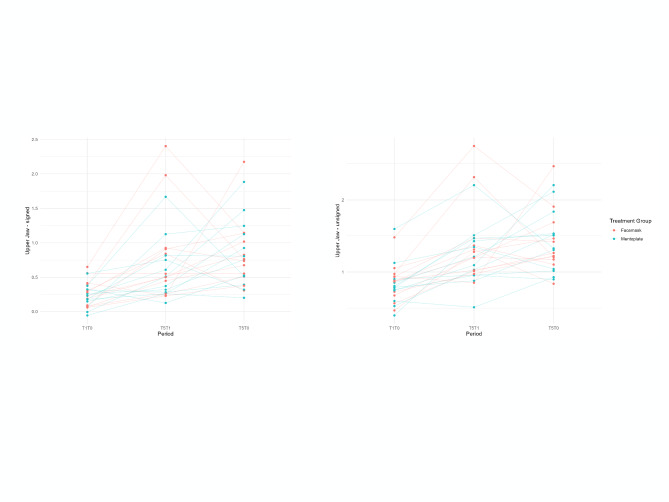
Maxillary changes. Scatter plot of maxillary changes. Left: signed values, Right: unsigned values

**Fig. 5:**
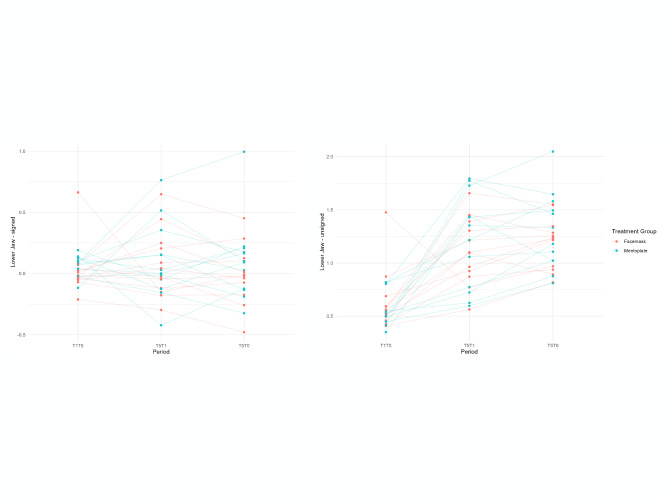
Mandibular changes. Scatter plot of mandibular changes. Left: signed values, Right: unsigned values

Both inter-observer (ICC: 0.95–0.98) and intra-observer (ICC: 0.93–0.98) measurements showed high reliability with no significant differences between observers. Mean follow-up time was 12 ± 1.59 months at T1 and 64.5 ± 5.21 months at T2 (Table 1). The skeletal changes are reported as both mean (signed) and absolute mean (unsigned) values. The 3D changes in maxillary and mandibular skeletal surfaces between the T0, T1 and T2 time points for both the FM and MP groups are shown in Table 2. A GEE model was fit to analyze the changes over time with type of treatment, age, gender and gonial angle as covariate. The difference in the FM group during active treatment period (T1–T0) is the reference group in the model (Table 3, Figs. 4 and 5). The signed mean values show both direction and magnitude of skeletal changes. A positive sign indicates anterior movement, while a negative sign shows posterior movement across three time periods (T0–T1, T0–T2, and T1–T2). Meanwhile, the unsigned mean values (absolute values) measure only the magnitude of changes, regardless of direction. Together, these measurements provide both qualitative (directional) and quantitative (magnitude) insights into skeletal changes.

**Table 2:** 3D volumetric changes

	T0–T1		T0–T2		T1–T2	
	Facemask	Mentoplate	Facemask	Mentoplate	Facemask	Mentoplate
*S mean*						
Mx	0.28 (0.20)	0.21 (0.17)	0.85 (0.50)	0.85 (0.51)	0.82 (0.69)	0.59 (0.45)
Md	0.03 (0.21)	0.07 (0.09)	− 0.01 (0.24)	0.10 (0.33)	0.06 (0.27)	0.10 (0.32)
*US mean*						
Mx	0.91 (0.23)	0.84 (0.30)	1.41 (0.43)	1.44 (0.44)	1.40 (0.57)	1.28 (0.36)
Md	0.66 (0.28)	0.57 (0.16)	1.19 (0.24)	1.30 (0.36)	1.11 (0.31)	1.22 (0.42)
*% forward growth*						
Mx	0.30	0.25	0.60	0.59	0.58	0.46
Md	0.05	0.12	− 0.01	0.08	0.05	0.08

S mean, signed mean; US mean, unsigned mean; Mx, maxilla; Md, mandible; mean (standard deviation); % forward growth, signed mean/unsigned mean

**Table 3:** GEE-model

	Mx signed				Mx unsigned				Md signed				Md unsigned			
Co-variate	Estimate	Std.err	95% CI	*p*-value	Estimate	Std.err	95% CI	*p*-value	Estimate	Std.err	95% CI	*p*-value	Estimate	Std.err	95% CI	*p*-value
Intercept	− 0.32	1.26	− 2.78 /2.14	0.80	− 0.68	1.61	− 3.84/ 2.49	0.67	− 2.47	1.08	− 4.58 / − 0.36	**0.02**	− 1.13	0.88	− 2.85 / 0.59	0.20
Mentoplate: T1–T0	− 0.10	0.08	− 0.25 / 0.05	0.18	− 0.11	0.10	− 0.30/0.08	0.25	− 0.02	0.08	− 0.18 / 0.14	0.81	− 0.15	0.12	− 0.38 / 0.09	0.22
T2–T1 (FM + MP)	0.54	0.17	0.22 / 0.86	**< 0.01**	0.55	0.12	0.31 / 0.78	**< 0.01**	− 0.04	0.08	− 0.19 / 0.11	0.63	0.47	0.13	0.22 / 0.72	**< 0.01**
T2–T0 (FM + MP)	0.58	0.13	0.32/0.83	**< 0.01**	0.53	0.12	0.30 / 0.76	**< 0.01**	0.04	0.10	− 0.15 / 0.23	0.67	0.55	0.11	0.33 / 0.77	**< 0.01**
Male	− 0.02	0.11	− 0.24 / 0.19	0.84	0.08	0.12	− 0.17 / 0.32	0.54	0.17	0.08	0.01 / 0.33	**0.03**	0.28	0.06	0.16 / 0.39	**< 0.01**
Age (months)	− 0.003	0.003	− 0.01 / 0.002	0.24	− 0.002	0.003	− 0.01 / 0.004	0.54	0.004	0.002	0.001 / 0.009	**0.02**	− 0.002	0.002	− 0.006 / 0.002	0.36
Gonial angle	0.01	0.01	− 0.01/0.03	0.35	0.01	0.01	− 0.01 / 0.03	0.17	0.02	0.01	< 0.01 / 0.03	**0.05**	0.02	0.01	0.004 / 0.03	**0.01**
Mentoplate:T2–T1	− 0.16	0.21	− 0.56/0.24	0.43	0.07	0.20	− 0.32/ 0.46	0.73	0.07	0.12	.− 0.16 / 0.30	0.54	0.18	0.18	− 0.18 / 0.54	0.33
Mentoplate: T2–T0	0.06	0.22	− 0.365 /0.48	0.80	− 0.13	0.15	− 0.42 / 0.16	0.37	− 0.01	0.14	− 0.28 / 0.27	0.96	0.19	0.15	− 0.11 / 0.48	0.22

Bold indicates *p*-value < 0.05

Differences are described in mm, interaction effect needs to be calculated, with the difference in the FM group during active treatment period (T1–T0) as reference group. Eg: differences in changes in the MP group T2–T0 compared to the FM group T2–T0 = (difference mentoplate: T1–T0) + (difference Mentoplate: T2–T0) = − 0.10 + 0.06 = − 0.04

Mx, maxilla; Md, mandible; Std.err, standard error; 95%CI, confidence interval; FM, facemask; MP, mentoplate;

When controlling for sex, age and gonial angle, both FM and MP treatments demonstrated similar effectiveness in maxillary advancement (signed values), with MP achieving slightly less advancement than FM (0.10 mm less at T1, *p* = 0.18). Initial forward movement was minimal (0.21–0.28 mm) but increased to 0.85 mm in both groups by the end of treatment. Notably, changes during follow-up (T2–T1) were 0.54 mm greater than those observed during active treatment (T1–T0, *p* < 0.01)). Total maxillary changes (unsigned values) increased from 0.9 mm initially to 1.4 mm at treatment completion, with no significant differences between groups (MP group exhibited 0.11 mm smaller changes at T1, *p* = 0.25 during active treatment).

Both treatments effectively controlled mandibular growth. Initial forward movement (mean signed values) was minimal in both groups (FM: 0.03 mm; MP: 0.07 mm), with MP showing slightly smaller changes (0.02 mm less, *p* = 0.81) during active treatment. By the end of treatment, the FM group exhibited slight backward movement (− 0.01 mm), while the MP group remained stable (0.1 mm). Final mandibular changes (unsigned values) were comparable between groups (*p* = 0.22). Signed mandibular changes during active treatment (T1–T0) were comparable to the signed changes observed during follow-up (T2–T1), *p* = 0.63. The unsigned changes during active treatment (T1–T0) were significantly smaller (0.47 mm) then during follow-up (T2–T1) (*p* < 0.01). Males experienced larger mandibular changes than females across both groups, with 0.28 mm greater absolute changes and 0.17 mm greater directional changes (*p* < 0.01 and *p* = 0.03, respectively). Additionally, patients with larger gonial angles demonstrated significantly greater mandibular volume changes. This effect was statistically significant in both signed (*p* = 0.05) and unsigned measurements (*p* = 0.01) (Table 3) (Supplementary material 1 for complete data).

### Ancillary analyses

No ancillary analyses were done.

### Harms

In our MP patient group, no plate loosening was observed. Although some issues with the anchor hooks (fracture or mucosal irritation) occurred, none led to treatment cessation. No mentoplate or screws required reinsertion. Fractures were managed by additional bending of the mentoplate, while mucosal irritation was resolved through optimized oral hygiene and the application of local chlorhexidine gel.

## Discussion

The primary aim of early intervention for Class III patients is to avoid orthognathic surgery when fully grown. This is done by promoting maxillary protrusion and restricting mandibular growth while mitigating unwanted outcomes like anterior shift of the upper dentition and vertical skeletal changes. Various strategies have been developed to achieve these objectives, such as facemask treatment or treatment with bone-anchors, which are used in conjunction with both intra- and extra-oral devices [10–24]. Treatment strategies with bone anchors have become increasingly more popular because they are often assumed to provide better skeletal control, and potential higher patient compliance [10, 38–40]. Our findings suggest that this theoretical advantage may not translate to better long term skeletal treatment effect.

Lack of prospective RCTs exist comparing the outcomes of different treatment protocols [5]. Only four prior RCTs have compared FM treatment to skeletal anchorage, and none of them report on long term results [4]. This scarcity of RCTs may stem from the challenges in convincing parents to enroll their children in a study where the treatment type is subject to randomization. To our knowledge, this study is the largest RCT with long term results comparing FM therapy with skeletal anchorage (MP) and the very first RCT to compare the long term effect of FM versus MP in combination with HH [4]. The assessment was conducted using a 3D approach which overcomes the limitations associated with lateral and frontal cephalograms, such as magnification error and distortion from structural superimposition.

Over 5 years, both FM and MP protocols produced similar skeletal treatment effects. These results align with Willmann et al.’s [41] short-term, retrospective 2D cephalometric study of 34 patients, which found comparable skeletal responses between HH with MP and FM. Our findings also support previous research showing that skeletal anchorage treatments do not produce significantly greater or faster skeletal changes than conventional methods [5, 12, 25]. However, direct comparisons with existing literature are limited, as most studies use 2D cephalometric analyses or linear and angular measurements based on cone beam CT scans rather than comprehensive 3D analysis.

Untreated Class III malocclusion tends to worsen with growth, characterized by excessive mandibular growth and a lack of maxillary catch-up growth [26, 42]. This growth pattern persists beyond adolescence, with mandibular growth continuing until about 17 years in females and after 18 years in males [43]. Analysis of jaw growth patterns show that forward growth (signed values) accounts for approximately half of the total upper jaw growth (unsigned values), while in the lower jaw only 7% of total growth has a forward vector at the 5-year follow-up in the MP group and even a negative (backward) growth pattern in the FM group. While these results suggest elastic traction effectively guided growth direction, the lack of a control group limits definitive conclusions. Surprisingly, the growth patterns established in the first year continued through the later follow-up phase, even after discontinuing Class III elastic traction. The upper jaw maintained approximately 50% forward growth, while the lower jaw showed only 5–8% forward growth. This is also reflected in the differences observed in the GEE model, where similar signed mandibular changes between active treatment (T1–T0) and follow-up (T2–T1) were present. However, unsigned changes were significantly larger during follow-up (*p* < 0.01). This suggests that successful early treatment creates better functional conditions for natural growth resulting in a comparable growth pattern in succeeding years in the lower jaw. Males exhibited greater mandibular growth than females, with 0.17 mm more forward growth (*p* = 0.03) and 0.28 mm more total growth (*p* < 0.01). These findings align with previous studies showing enhanced mandibular growth in male Class III patients [44]. A large gonial angle, which indicates vertical growth pattern, correlated with mandibular growth in our patients for both signed (*p* = 0.05) and unsigned (*p* = 0.01) changes. While some suggest that a large initial gonial angle may predict poor treatment outcomes, current evidence supporting this claim is limited [45, 46]. Our findings demonstrate that increased gonial angles correlate with greater mandibular changes, though the impact on treatment outcomes remains uncertain.

A treatment protocol involving MP offers several potential advantages as a bone anchor compared to other options. It can be inserted in the lower jaw before the eruption of the mandibular canines, as it is fixed to the bone in the chin area below the mandibular incisors (Fig. 1). This allows for earlier initiation of interceptive treatment. It has been suggested that early Class III treatment before the age 10 provides better results [29, 47–52]. Additionally, the placement of the screws away from the tooth roots ensures the design safety, significantly reducing the risk of injuring adjacent teeth. In our study, no loosening of the MP was observed. This is likely due to its one-piece construction, which allows for simultaneous traction on both sides and a resultant force vector directed towards the mandibular bone, preventing plate loosening. In contrast, other bone anchor designs have a higher potential for loosening or interfering with the roots of adjacent teeth [27]. Some minor issues like plate fracture and mucosal irritation were observed in our study but did not lead to treatment cessation. Importantly, placing MP and palatal screws for the HH does not require an incision with periosteal stripping in the upper jaw, resulting in less swelling and postoperative discomfort compared to traditional bone anchors used in BAMP protocols. From a surgical perspective, MP is the anchor of choice due to its lower risk of loosening, fewer potential complications related to adjacent teeth, and reduced postoperative discomfort for the patient.

### Limitations

The results were based on a 12-patient-per-group observation. Individual variation in growth patterns and genetic predisposition for class III malocclusion may significantly influence treatment outcomes, regardless of the intervention chosen. Our sample size, while statistically adequate, may not fully represent the spectrum of genetic variability in Class III patients. Significant patient dropout occurred at the five-year follow-up, also in the MP group. This was unexpected since MP patients typically return for hardware removal. Though all MP were eventually removed, the varying removal times extended beyond T2, leading to these patients’ exclusion to avoid bias. Our study focused on comparing skeletal effects between the two protocols, rather than evaluating Class III malocclusion correction. While this approach may be less clinically intuitive, it provides objective quantification of skeletal changes. Our study analyzed complete 3D skeletal models. Future research should decompose the structures into distinct anatomical segments to provide a more detailed depiction of skeletal changes. This segmented approach would allow detailed analysis of dental, palatal, zygomatic, and soft tissue changes. Additionally, future studies should evaluate patient compliance and pain perception associated with MP. This RCT focused specifically on appliance design and mechanics and its findings challenge the common assumption that bone anchors provide superior results despite being more invasive.

### Generalisability

Our results are primarily applicable to patients in the mixed dentition phase (average 9.7 ± 1.3 years) with moderate skeletal discrepancies. The findings may not extend to patients with severe skeletal class III malocclusions or those at different developmental stages.

As this trial was conducted in a single center with experienced clinicians, results might vary in different clinical settings. We used a standardized Alt-RAMEC protocol and force system. Different expansion protocols or force magnitudes might yield different outcomes.

### Interpretation

It’s challenging to distinguish between natural growth changes and treatment effects in Class III cases. Clinicians cannot reliably predict which patients will respond well to early intervention [45, 46]. Patients and parents should be informed that 20–30% of cases may ultimately require orthognathic surgery, even after interceptive treatment [7, 48, 52–55]. The individual’s growth pattern may be the most critical factor influencing outcomes, rather than the specific treatment technique employed. Early treatment success should be monitored closely during the first 6–12 months, as positive treatment effect potentially provides a better myofunctional situation for further growth. For those who do not respond well, it’s often best to delay treatment until the patient has fully matured, at which point orthognathic surgery becomes the best option. Since both treatment protocols yield comparable skeletal effects in the long term, orthodontists may regard them as equally viable options for early class III treatment. This is particularly relevant for clinical decision making, as it suggests that the choice between FM and MP should be based on individual patient factors rather than presumed mechanical advantages. This consideration may encompass patient-specific factors such as compliance challenges or anatomical constraints.

## Supplementary Information

Supplementary Material 1.

Supplementary Material 2.

## Data Availability

The authors confirm that the data supporting the findings of this study are available within the article and its supplementary materials.
