# Correction to: Long-term three-dimensional skeletal effects of hybrid hyrax with facemask versus mentoplate in growing Class III patients: a randomized controlled trial

**DOI:** 10.1186/s40510-025-00574-2

**Published:** 2025-07-17

**Authors:** Joeri Meyns, Jindanil Thanatchaporn, Sohaib Shujaat, Constantinus Politis, Prog Orthod

**Affiliations:** 1 Ziekenhuis Oost-Limburg, Genk, Belgium; 2 KU Leuven, Leuven, Belgium; 3 Chulalongkorn University, Bangkok, Thailand; 4 King Abdullah International Medical Research Center, Jeddah, Saudi Arabia; 5 Karolinska Institutet, Stockholm, Sweden

**Correction: Progress in Orthodontics (2025) 26:14**

10.1186/s40510-025-00561-7

Following publication of the original article [1], the authors identified errors in the Introduction Objective section and captions of Figs. 1 and 3; Table 1 which were missing that these were reused from another article by Meyns et al. [55].

The Introduction with sub-section Objective reads:

**Objective**

This prospective RCT aimed to compare the 5-year longterm 3D skeletal effects of HH + FM and HH + MP treatment protocols in growing class III patients using low dose computed tomography (CT) scans.

The Introduction with sub-section Objective should read:

**Objective**

This prospective RCT aimed to compare the 5-year longterm 3D skeletal effects of HH + FM and HH + MP treatment protocols in growing class III patients using low dose computed tomography (CT) scans. A previously published study by the same authors was published in the European Journal of Orthodontics, which reported on the 2D cephalometric changes of the same patient cohort [55].

The incorrect Fig. 1 caption reads:

**Fig. 1** **A** Extra-oral view Facemask (FM), **B** Hybrid hyrax FM, **C** Mentoplate (MP) secured between impacted canines, **D** Hybrid hyrax MP

The correct Fig. 1 caption should read:

**Fig. 1** **A** Extra-oral view Facemask (FM), **B** Hybrid hyrax FM, **C** Mentoplate (MP) secured between impacted canines, **D** Hybrid hyrax MP. Reproduced from Meyns et al. [55] with authors’ permission

The incorrect Fig. 3 caption reads:

**Fig. 3** Participants flow

The correct Fig. 3 caption should read:

**Fig. 3** Participants flow. Reproduced from Meyns et al. [55] with authors’ permission

The incorrect Table 1 caption reads:

F/M, Female/Male; Follow-up (T2), mean follow-up time in months, p-value (unpaired two-sided samples t-test). Baseline comparisons are provided for descriptive purposes only, to confirm group equivalence following randomization, and should not be interpreted as inferential statistical analyses.

The correct Table 1 caption should read:

F/M, Female/Male; Follow-up (T2), mean follow-up time in months, p-value (unpaired two-sided samples t-test). Baseline comparisons are provided for descriptive purposes only, to confirm group equivalence following randomization, and should not be interpreted as inferential statistical analyses. Reproduced from Meyns et al. [55] with authors’ permission.

The Introduction Objective section and Figs. 1 and 3; Table 1 captions have been updated above and the original article [1] has been corrected.
